# The *SlDOG1* Affect Biosynthesis of Steroidal Glycoalkaloids by Regulating GAME Expression in Tomato

**DOI:** 10.3390/ijms24043360

**Published:** 2023-02-08

**Authors:** Xuecheng Zhao, Yueran Zhang, Jun Lai, Yuan Deng, Yingchen Hao, Shouchuang Wang, Jun Yang

**Affiliations:** 1 Hainan Yazhou Bay Seed Laboratory, Sanya Nanfan Research Institute of Hainan University, Sanya 572025, China; 2 College of Tropical Crops, Hainan University, Haikou 570228, China

**Keywords:** glycoalkaloid metabolism, GWAS, DOG1-like transcription factor, steroidal glycoalkaloids, tomato

## Abstract

Steroidal alkaloids (SAs) and steroidal glycoalkaloids (SGAs) are common constituents of plant species belonging to the *Solanaceae* family. However, the molecular mechanism regulating the formation of SAs and SGAs remains unknown. Here, genome-wide association mapping was used to elucidate SA and SGA regulation in tomatoes: a SlGAME5-like glycosyltransferase (Solyc10g085240) and the transcription factor SlDOG1 (Solyc10g085210) were significantly associated with steroidal alkaloid composition. In this study, it was found that rSlGAME5-like can catalyze a variety of substrates for glycosidation and can catalyze SA and flavonol pathways to form *O-glucoside* and *O-galactoside* in vitro. The overexpression of SlGAME5-like promoted α-tomatine, hydroxytomatine, and flavonol glycoside accumulation in tomatoes. Furthermore, assessments of natural variation combined with functional analyses identified SlDOG1 as a major determinant of tomato SGA content, which also promoted SA and SGA accumulation via the regulation of GAME gene expression. This study provides new insights into the regulatory mechanisms underlying SGA production in tomatoes.

## 1. Introduction

Steroidal alkaloids (SAs) and steroidal glycoalkaloids (SGAs) are nitrogen-containing toxic compounds, predominantly found in plant species of the *Solanaceae* and *Liliaceae* families [1], with about 1350 species documented to contain SAs to date [2]. The most common steroidal alkaloids in *Solanum tuberosum* L., α-tomatine and α-chaconine, were the main steroidal alkaloids in *Solanum tuberosum* L., accounting for about 90% of the total steroidal alkaloids (SAs and SGAs) [3,4]. Although steroidal alkaloids are beneficial to plants via their involvement in disease resistance and anti-herbivory responses [5,6], due to their toxic effects in humans, some steroidal alkaloids are considered antinutritional factors [7]. In tomatoes, nearly 100 steroidal alkaloids have been detected, with α-tomatine and dehydrotomatine being the main toxic substances in green tissues [2,8]. When ingested, toxic SAs can destroy cell membrane components, leading to gastrointestinal dysfunction and nervous system effects; SAs show concentration-dependent toxicity, which can be fatal in severe cases [9]. Note that α-tomatine is also the main component of steroidal glycoalkaloids in tomatoes; α-tomatine is distributed in the leaves, flower buds, and green fruit tissues of tomato plants [10,11,12].

Recently, a group of galactosyltransferase (GAME) genes was found to participate in SGA production in tomato metabolism, such as GAME1, GAME17, GAME18, and GAME2 [9]. During tomato fruit ripening, a series of modifications convert tomatidine into the non-bitter and non-toxic esculeoside A [9,13,14]. In tomatoes, *SlGAME1* was the first gene reported to synthesize α-tomatine, which mainly performs multi-step glycosylation in the SGAs metabolic pathway [2]. SlGAME31, a dioxygenase dependent on 2-oxoglutarate, catalyzes the conversion of α-tomatine to hydroxytomatine [15]. Later in the process, SlGAME5 catalyzes the formation of esculeoside A from acetoxy-hydroxytomatine [14]. In addition, *SlGAME5* has gained a stop mutation, affecting the evolution of steroidal alkaloid content [16].

The study of natural genetic variation has become a useful approach for investigating the molecular functions of pivotal genes [17,18]. Genome-wide association studies (GWAS) and metabolome-based genome-wide association studies (mGWAS) have provided considerable insight into the extent of natural metabolomic variation, as well as its genetic and biochemical controls, in tomatoes and rice [16,19,20,21]. To satisfy marketing and economic demands, tomato breeders are constantly working to improve the crop [14,16,22,23]. 

The *DELAY OF GERMINATION 1* (DOG1) gene belongs to the bZIP family of transcription factors. In *Arabidopsis thaliana* and rice, DOG1 is believed to affect seed dormancy and flowering time [24,25,26]. In *Nicotiana tabacum*, NtabDOG1L regulates plant development and enhances drought resistance [27]. Furthermore, AtDOG1 regulates ABI5 activity to affect the expression of hundreds of gene, which implies that DOG1’s function is not limited to seed dormancy [28]. 

Here, we identified a *SlGAME5* homologous gene involved in the biosynthesis of α-tomatine; this gene is likely important for reducing bitterness. Furthermore, using an mGWAS approach based on natural variation, we found SlDOG1 to be a primary determinant of SGA content in tomato leaves and fruits.

## 2. Results

### 2.1. Metabolic Profiling of SA and SGA Compounds in Tomato at Different Developmental Stages

**Figure 1:**
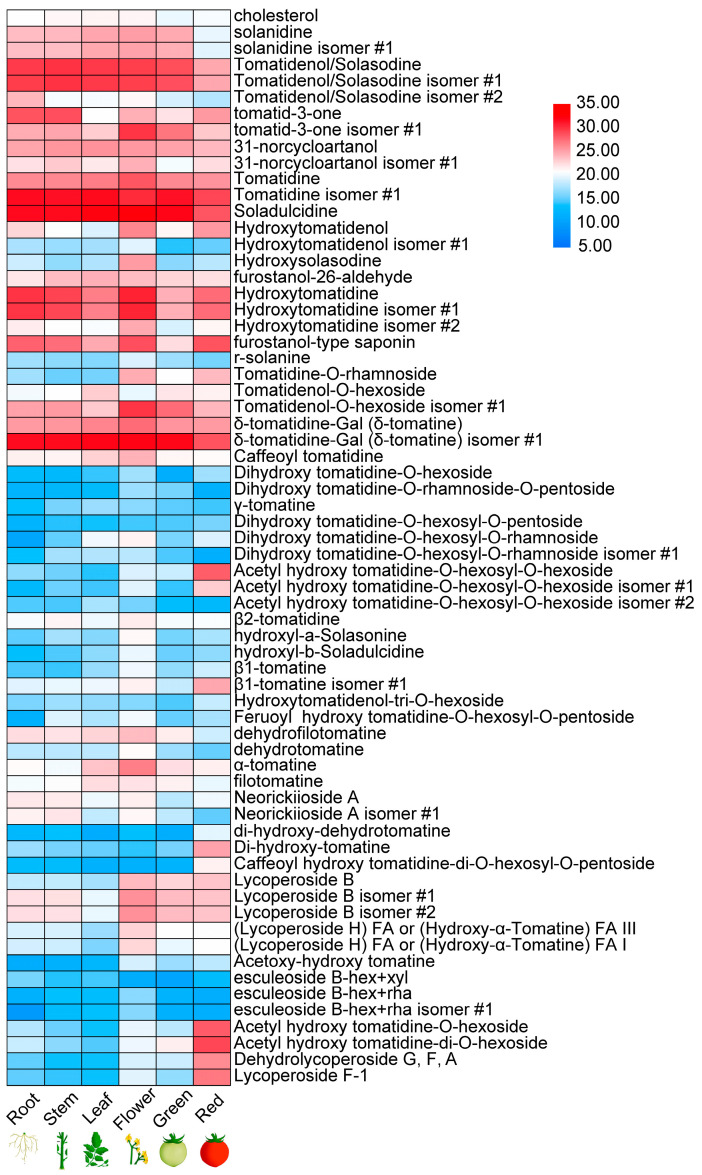
Metabolite contents during fruit development and ripening: diversity of SAs present in different tissues in tomato plants. isomer #: represents an isomer.

To characterize the occurrence of SAs and SGAs across developmental stages in tomatoes (CV Micro-Tom), six major growth stages were examined in a UPLC-qTOF-MS analysis. A total of 48 metabolites, including tomatidine, α-tomatine, hydroxytomatine, dehydrotomatine, and lycoperoside B, were associated with different tissues, while the remaining substances were putatively identified as their isomers (Figure 1; Supplemental Data S3). Seven SGAs were associated with ripe (red) fruits, including acetyl hydroxy tomatidine-O-hexosyl-O-hexoside, di-hydroxy-tomatine, lycoperoside B, acetyl hydroxy tomatidine-O-hexoside, acetyl hydroxy tomatidine-di-O-hexoside, dehydrolycoperoside G, dehydrolycoperoside F or dehydroescoleoside A, and lycoperoside F-1. However, tomatidenol, tomatidine, and dehydrotomatine were only present at low levels in red fruits (Figure 1). In flowers, α-tomatine, lycoperoside B isomer #1, and lycoperoside B isomer #2 were found to accumulate (Figure 1). In addition, accumulation patterns for SAs and SGAs in roots and stems were very similar (Figure 1). These results suggest that tomatidenol, tomatidine, and α-tomatine catalyze the formation of non-bitter and non-toxic esculeoside A, which accumulates in ripening fruits.

### 2.2. Identification and Analysis of SlGAME5-like and SlDOG1 via GWAS

**Figure 2:**
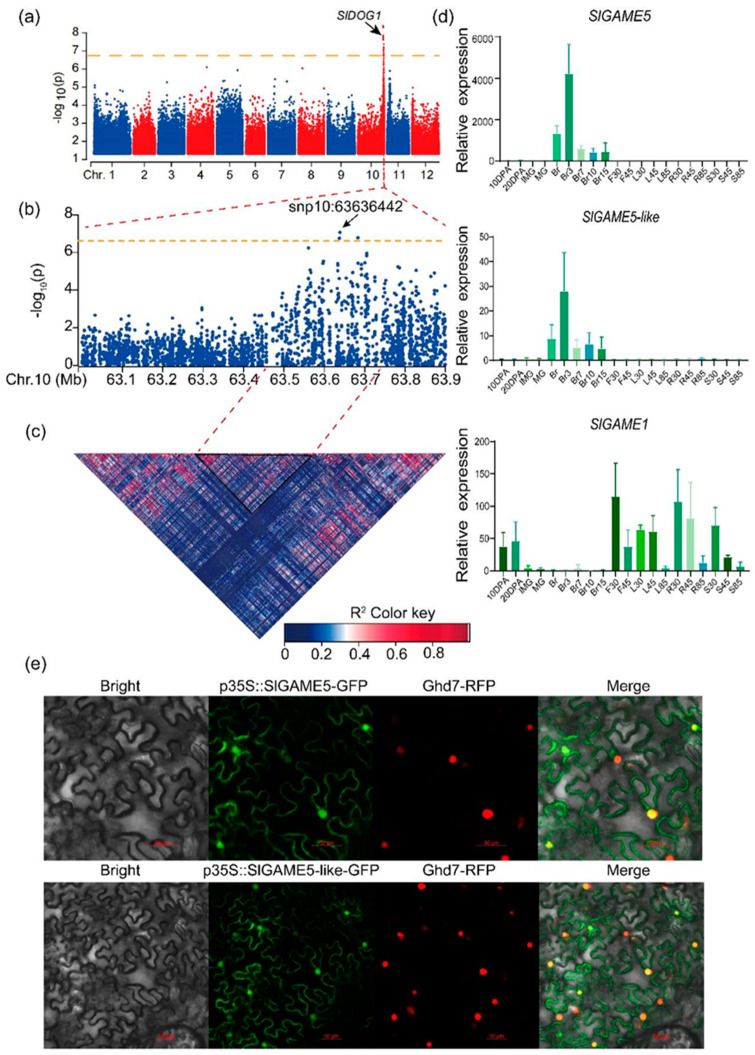
Identification and bioinformatic analysis of *SlGAME5-like* via genome-wide association mapping: (**a**) Manhattan plot showing the location of *DOG1*, as identified using a GWAS of the glucoside metabolite content of steroidal alkaloids. (**b**) Gene model of *SlDOG1*. (**c**) A representation of pairwise R^2^ (a measure of LD) among all polymorphic sites within the ~200 kb region surrounding the most significant GWAS SNP 10:63636442. The color of each cell corresponds to its R^2^ value, as described in the legend. (**d**) Transcriptome data showing the relative expression of *SlGAME5*, *SlGAME5-Like* and *SlGAME1* in different growth stages. Twenty samples from three key developmental stages were collected for metabolic profiling and RNA-seq. The *x*-axis indicates the sample harvest date (DPG). Leaf (L), root (R), stem (S), bud (F30), and flower (F45) samples were harvested at the bud stage (30 DPG), flowering stage (45 DPG), and breaker stage (85 DPG). Fruit samples were harvested at the following stages: 10 DPA, 20 DPA, immature green (IMG), mature green (MG), breaker (Br), three days post-breaker (Br3), Br7, Br10, and Br15 [12]. (**e**) Nuclear localization of GFP-SlGAME5 and GFP-SlGAME5-like fusion proteins in the leaf epidermal cells of *Nicotiana benthamiana*. Bar = 50 µm.

In order to better understand the genetic basis of SGA variation in natural tomato populations, based on previous studies [16,23,29], we performed a genome-wide association analysis using α-tomatine as a phenotype (GWAS) (based on 298 tomato germplasms) (Supplemental Data S4). α-tomatine is a key substance for the conversion of toxic and bitter substances to non-bitter substances in SGAs [2,13]. The most significant single nucleotide polymorphism (SNP) site is present at the end of chromosome 10 (Figure 2a) and is localized to a second exon named the SlDOG1 transcription factor. Based on patterns of linkage disequilibrium (LD) decay, we retrieved genes falling within 50 kb of the leading (i.e., most significant) SNP (10_63636442) and considered their functions to select candidate genes for tomatidine (Supplemental Data S5). In this LD block, we found seventeen candidate genes (Figure 2b,c). In order to further narrow the scope of candidate genes, we summarized and further inferred the metabolic reactions related to tomatidine in tomato fruit. We found that the upstream and downstream metabolic reactions of tomatidine are glycosylation. Surprisingly, two of these seventeen potential candidate genes are called glycosyltransferases; one glycosyltransferase was SlGAME5, and the other is named SlGAME5-like. Glycosidation is a crucial reaction for tomatidine to form non-toxic esculeoside A. We infer that these genes may together participate in esculeoside A biosynthesis.

In a phylogenetic tree, SlGAME5-like protein and SlGAME5 occurred in the same branch (Figure S1). SlGAME5 is a glycosyltransferase with galactose catalytic activity, implying that SlGAME5-like may have a similar function of catalyzing steroidal alkaloids to form glycosides. Aligning SlGAME5-like with SlGAME1, SlGAME5, and other uridine 5′-diphospho-glucuronosyl transferases (UGTs) revealed a conserved PSPG box in the C-terminus (Figure S2). The expression of *SlGAME5* and *SlGAME5-like* was higher in the fruit ripening stage than in other developmental stages (Figure 2d; Supplemental Data S6). However, *SlGAME1* was highly expressed in green tissues (Figure 2d; Supplemental Data S6). This suggests that SlGAME5 and SlGAME5-like mainly catalyze SAs in red fruits, while SlGAME1 catalyzes SAs in green leaves. Interestingly, SlDOG1 has a similar expression pattern to SlGAME5 and SlGAME5-like. Genes with similar expression patterns may have similar functions. Therefore, combined with mGWAS and expression profile analysis, it is speculated that SlDOG1 may be involved in regulating the expression of SlGAME5 and SlGAME5-like (Figure 2d; Figure S4). Analyses of gene structure and promoter *cis*-regulatory elements identified many transcriptions factor binding elements in the promoter regions of both genes (Figure S3). In order to determine the specific position of SlGAME5 and SlGAME5-like gene expression proteins in cells, the fusion expression vector was constructed, and the subcellular localization level was analyzed using the transient expression method of tobacco epidermal cells. Using confocal microscopy, it was observed that there were obvious signals in the nucleus and cell membrane, indicating that SlGAME5 and SlGAME5-like genes were mainly localized in the nucleus and cell membrane (Figure 2e).

### 2.3. Characterization of a Putative SlGAME5-like Protein

**Figure 3:**
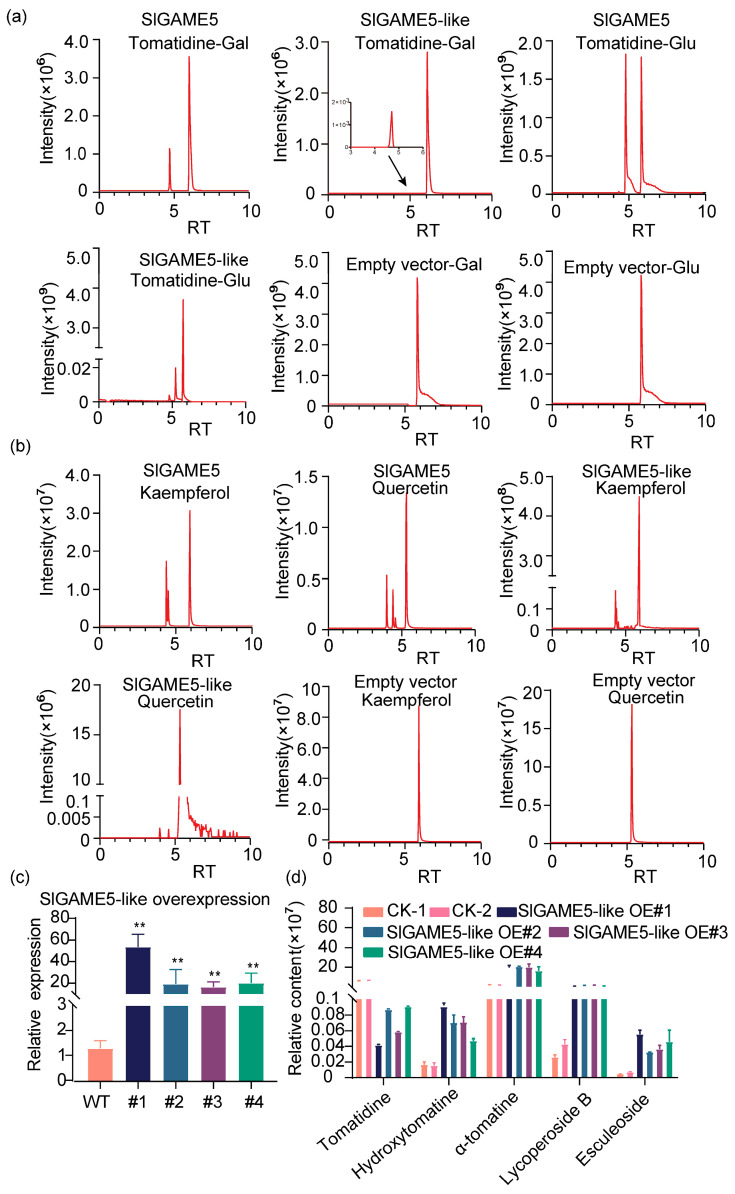
Enzymatic assays of recombinant GAME gene products in vitro. (**a**,**b**): Analysis of recombinant SlGAME5 and GFP-SlGAME5-Like enzymatic reaction products. (**c**): *SlGAME5-like* expression levels in three independent transgenic lines as determined by real-time PCR analysis. (**d**): SlGAME5-like plants showed increased accumulation of lycopene and its precursor. The absolute concentrations of lycopene and its precursor were determined in fully expanded leaves from four-week-old seedlings using UPLC-qTOF-MS and compared with those of wild-type plants of the same age. Data were from three independent experiments and expressed as means ± SD. (*n* = 3). The differences were analyzed in two tailed comparisons with the control, and ** *p* < 0.01 in the student’s *t*-test.

To investigate the enzymatic activity of SlGAME5 and SlGAME5-like, we expressed both genes in *E. coli*, and the molecular weights of the recombinant proteins PGEX-6P-1-SlGAME5 and PGEX-6P-1-SlGAME5-like were 79.54 kDa and 79.14 kDa, respectively, as identified using sodium dodecyl sulfate polyacrylamide gel electrophoresis (SDS−PAGE) (Figure S4). To test the substrate specificity of SlGAME5 and SlGAME5-like, two sugar donors (UDP-glucose and UDP-galactose) and tomatidine were tested as substrates (Figure S5). The enzyme assay results showed that SlGAME5 and SlGAME5-like both catalyze tomatidine to form α-tomatine with UDP-glucose and UDP-galactose as the sugar donors in vitro (Figure 3a); they also catalyze kaempferol and quercetin to form their own glucosides (Figure 3b). 

Furthermore, the SlGAME5 catalytic efficiency was low for tomatidine but high for quercetin, with a *Km* value for quercetin of 17.50 μM (Figure S6). However, for SlGAME5-like, there was no difference in catalytic efficiency between the two substrates: *Km* values for quercetin and tomatidine were 33.00 μM and 40.00 μM, respectively (Figure S6). Next, to investigate the function of *SlGAME5-like*, we overexpressed *SlGAME5-like* in tomatoes. Using UPLC-qTOF-MS, SA-targeted profiling was performed to determine how SA profiles differed between the wild type and overexpression lines. Levels of tomatidine were reduced in the overexpression lines, but α-tomatine, hydroxymatine, and flavonol glycosides increased (Figure 3c,d; Figure S7). Similarly, lycopene and its precursor in fruits detected at the breaking stage also have a similar trend of change (Figure S8). These results suggest that SlGAME5-like represents a glycosyltransferase (similar to SlGAME1) that is expressed in red fruits and which play an important role in improving tomato flavor. 

### 2.4. Leaves of SlDOG1-OX Plants Exhibit Altered Levels of SAs and SGAs

**Figure 4:**
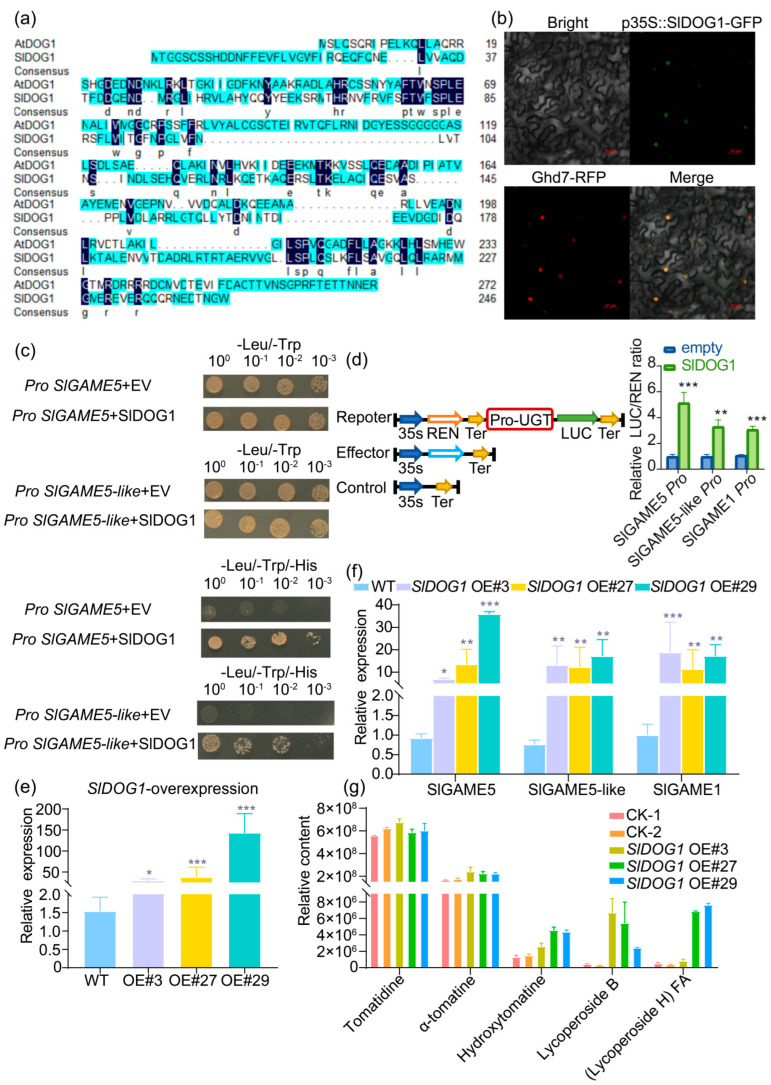
SlDOG1 interacts with SlGAME in vivo and in vitro. (**a**) Multiple sequence alignment of SlDOG1 and its homologs. (**b**) Nuclear localization of GFP-SlDOG1 fusion proteins in the leaf epidermal cells of *Nicotiana benthamiana*. Bar = 50 µm. (**c**) A yeast one-hybrid assay showing interactions between SlDOG1 and *SlGAME5*, *SlGAME5-like*. (**d**) Dual luciferase reporter assay showing how SlDOG1 can induce the activity of a common promoter of *SlGAME1-like* genes. (**e**) *SlDOG1* expression levels in three independent transgenic lines as determined by real-time PCR analysis. (**f**) The expression levels of *SlGAME5*, *SlGAME5-like*, and *SlGAME1* in SlDOG1-overexpressing plants as determined via real-time PCR. (**g**) SlDOG1-OE plants showed heightened accumulation of lycopene and its precursor. The absolute concentrations of both were determined in fully expanded leaves from four-week-old seedlings using UPLC-qTOF-MS and compared with those of wild-type plants of the same age. CK-1, CK-2: wild-type plants of the same age. Data were from three independent experiments and expressed as means ± SD. (n = 3). The differences were analyzed in two tailed comparisons with the control, and * *p* < 0.05; ** *p* < 0.01; *** *p* < 0.001 in the student’s *t*-test.

To investigate the function of SlDOG1 in tomatoes, its sequence was aligned to similar proteins, and a phylogenetic analysis was performed. SlDOG1, SaDOG1, HvDOG1L1, HvDOG1L2, TaDOG1L4, and AtDOG1 all had conserved region 1, region 2, and region 3 elements (Figure 4a; Figure S9). In the phylogenetic tree, SlDOG1 was closely related to the DOG1 transcription factor DOG1L5 group (Figure S10a). Analysis of the *SIDOG1* promoter identified ABRE, BOX4, and TCA cis-elements (Figure S10b). Moreover, *SlDOG1* was highly expressed in the fruit ripening stage (Figure S11; Supplemental Data S3). Thus, SlDOG1 has a potential regulatory effect on *SlGAME5* and *SlGAME5-like*. Most transcription factors function in the nucleus. SlDOG1 as a transcription factor and GFP-S1DOG1 fusion protein signal were observed in the nucleus of tobacco epidermal cells (Figure 4b). SlDOG1 bound to the promoters of both *SlGAME5* and *SlGAME5-like* (Figure 4c). Using a dual luciferase reporter gene system, a PH2GW7 vector (for proSl-GAMEs) and a pEAQ-HT-D vector (for SlDOG1) were constructed and transferred into tobacco for promoter activation experiments (Figure 4d). Transactivation assays revealed that SlDOG1 enabled the activation of *SlGAME5*, *SlGAME5-like*, and *SlGAME1* expression (Figure 4d). 

To describe the role of SlDOG1 in SGA biosynthesis, we generated transgenic tomato lines in which *SlDOG1* was overexpressed (SlDOG1-OE). A real-time PCR analysis of leaf tissue revealed the higher expression of *SlDOG1* in the SlDOG1-OE lines (Figure 4e). Likewise, *SlGAME5*, *SlGAME5-like*, and *SlGAME1* were also significantly up-regulated in these lines (Figure 4f). Using UPLC-qTOF-MS, SA and SGA profiling was carried out on tomato leaf extracts. In leaves from the SlDOG1-OE lines, the levels of hydroxytomatine, lycoperside B, and (lycoperside H) FA were 2–4-fold, 10–20-fold, and 10–15-fold higher, respectively, as compared to leaves of wild-type plants (Figure 4g). In addition, SlDOG1 also promoted tomatidine and α-tomatine accumulation (Figure 4g). The accumulation of lycopene and its precursor in the fruit of SlDOG1-overexpressing plants at the breaking stage also showed a similar trend (Figure S12). This suggests that SlDOG1 promotes SA and SGA synthesis by regulating *SlGAME5*, *SlGAME5-like*, and *SlGAME1* expression.

### 2.5. A Variant of SlDOG1 Is Associated with SGA Content in Tomato Fruits 

A genome-wide association analysis was performed using metabolomic data; for tomatidine, *SlDOG1* was identified as a candidate gene. The most-significant SNP (10_63636442) represented a non-synonymous mutation (C-G) located in the second exon of *SlDOG1*; this mutation resulted in a threonine (Thr) to methionine (Met) substitution (Figure S13). Fruit weight and size were key traits in the early domestication and improvement of tomato, and many genes related to weight were, therefore, co-selected; due to hitchhiking effects, genes underlying SGAs were also synergistically selected during human selection for tomato fruit size and weight [16,23,29].

**Figure 5:**
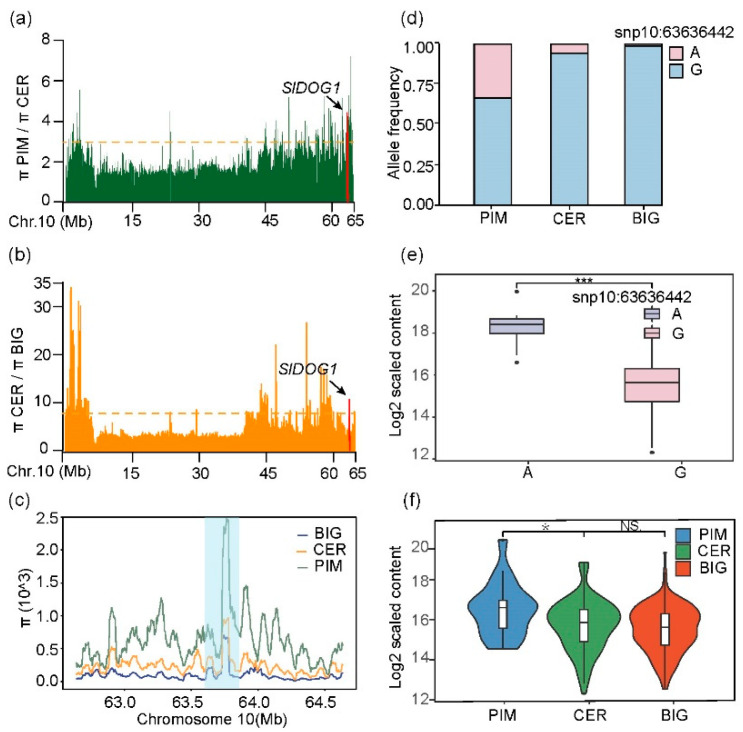
Analysis of variation in SlDOG1 in tomato. (**a**–**c**): Nucleotide diversity ratios between PIM and CER (**a**) and between CER and BIG (**b**) on chromosome 10. The orange dashed horizontal lines indicate the top 10% threshold for all of chromosome 10 (1.82 pPIM/pCER for domestication and 4.27 pCER/pBIG for improvement). The red color indicates the position of SlDOG1 in selective sweeps. (**d**): Allele frequency distribution for two *SlDOG1* major alleles (A or G) in PIM, CER, and BIG. (**e**): Analysis of natural variation associated with *SlDOG1* in tomato. Box plots of tomatidine content for each *SlDOG1* allele. In the box plots, the middle line indicates the median, the box indicates the interquartile range, the whiskers indicate the minimum and maximum values, and the outer dots indicate any outliers. Significant differences were identified using Student’s *t*-tests. (**f**): Violin plots of tomatidine content in PIM, CER, and groups. Data were from three independent experiments and expressed as means ± SD. (*n* = 3). The differences were analyzed in two tailed comparisons with the control, and * p < 0.05; *** p < 0.01; ns: no significant difference, *p* > 0.05 in the student’s *t*-test.

To elucidate the evolutionary history of the *SlDOG1* locus, we performed a selective sweep analysis. The chromosomal segment containing *SlDOG1* was found within a sweep region for the domesticated and improved tomato accessions (Figure 5a,b); additionally, BIG, CER, and PIM showed significant polymorphisms in this region (Figure 5c). A haplotype analysis showed that, relative to the reference genome (modern cultivated tomato Heinz 1706), the frequency of haplotype 1 (A) was significantly higher in wild gooseberry tomato (PIM) than in early domesticated tomato varieties (CER) and modern tomato cultivars (BIG) (Figure 5d). The tomatidine content of haplotype 1 (A) was also significantly higher than that of haplotype 2 (G) (Figure 5e). Previous studies have shown that a higher steroidal alkaloid content reduces tomato flavor and quality and that the steroidal alkaloid content in cultivated tomatoes is significantly lower than in wild tomatoes [16,23,29]. Therefore, we speculate that the snp:10_63636442 mutation affected the activity of *SlDOG1*; the synergistic selection of snp:10_63636442 during breeding efforts then led to variation in solanine content among different subgroups of tomato. However, the high-steroidal alkaloid type was lost during the tomato breeding process, while the low-steroidal alkaloid type was selected and retained, eventually increasing in frequency. This scenario is supported by our statistical analysis of tomatidine content across tomato accessions. Tomatidine content varied among tomato subgroups, with the highest levels seen in PIM, followed by CER and BIG (Figure 5f). These results suggest that human selection of fruit size and weight in tomatoes was accompanied by linked effects on other genes and metabolites. 

## 3. Discussion

**Figure 6:**
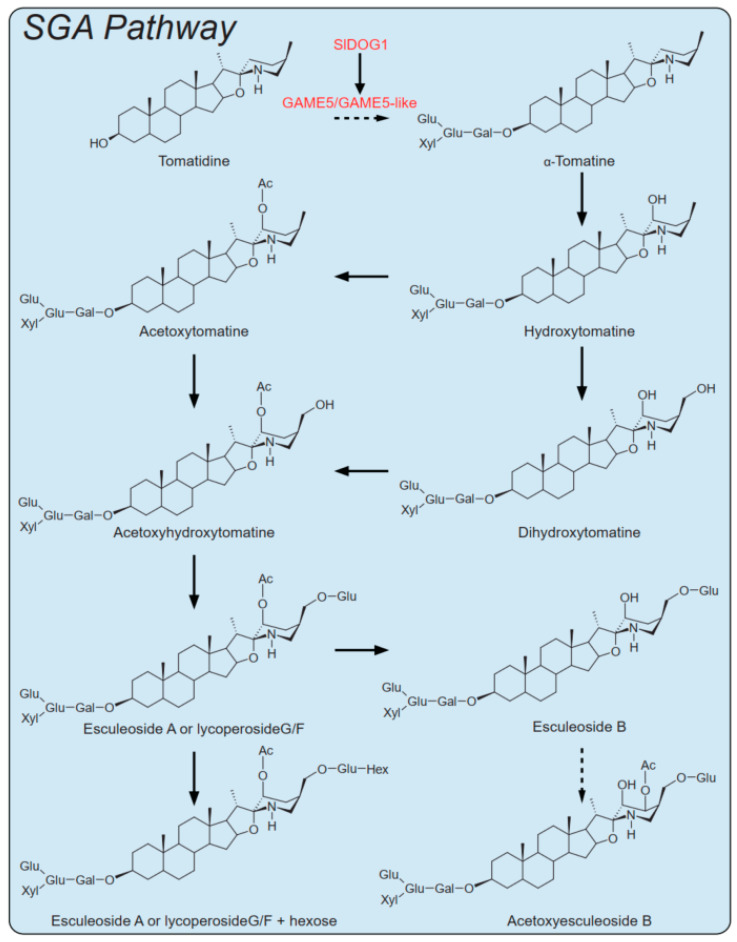
Core metabolic pathways: A simplified scheme of the core SGA metabolic pathway during tomato fruit development and ripening, which includes hydroxylation, acetylation, and glycosylation steps. During ripening, the bitter-tasting α-tomatine is converted to the non-bitter esculeoside A. Ac, acetoxy; Gal, galactose; Glc, glucose; Xyl, xylose.

In this study, we identified a GAME5-like glycosyltransferase, a GT-type enzyme that uses UDP-Gal and UDP-Glu to catalyze tomatidine to form α-tomatine in vitro. In addition, a transcription factor, SlDOG1, was identified via GWAS and mGWAS analyses; genetic and biochemical analyses suggested that SlDOG1 promotes SA and SGA biosynthesis in tomatoes (Figure 6). This discovery provides important insight into the SA and SGA biosynthetic pathways, and sheds light on the significance of glycosylation in the prevention of self-toxicity in plants.

### 3.1. Accumulated Diversity of SAs and SGAs in Different Developmental Stages

To improve tomato quality and satisfy marketing and economic demands, recent research has focused on metabolic profiling, especially for the biosynthesis of SAs and SGAs [8,30,31,32]. In a recent comprehensive study, metabolic profiling of 20 major tomato tissues and growth stages revealed the presence of α-tomatine and dehydrotomatine derivatives in green tomato tissues, including buds and flowers; in the fruiting stage, lycoperoside G/F and esculeoside A were the main metabolites [2,12]. 

Here, about 80 different SAs and SGAs were examined, and several groups of related chemicals were identified across six tomato tissues. Many SA and SGA isomers were identified in different tissues, including tomatidenol, hydroxytomatine, and lycoperside (Figure 1). Metabolites showed tissue specificity; for example, α-tomatidine primarily accumulated in flowers, while acetylhydroxytoamtidine-O-hexosyl-O-hexoside accumulated in red fruits. Notably, tomatidine and dehydrotomatine were the most common toxic substances identified in green tissues (leaves, stems, and green fruits); in ripe (red) fruits, the non-bitter and non-toxic esculeoside A and lycoperoside F were predominant. These results imply that SAs and SGAs vary across life stages and tissues, showing diversity over time. Furthermore, different tissues contain sets of enzymes to form non-bitter and non-toxic esculeoside A or lycoperoside F, which both accumulate in ripening fruits.

### 3.2. The Importance and Functional Diversity of the GAME Gene Family in Plants

During fruit ripening in tomatoes, many toxic substances (e.g., tomatidine and α-tomatidine) undergo a set of glycosylation, hydroxylation, and acylation reactions that produce non-bitter and non-toxic SGAs [2,13,14]. In these reaction pathways, the first step involves glycoxidation. SlGAME1 catalyzes tomatidine to form α-tomatine using UDP-glucose or UDP-galactose as sugar donors in vitro; GAME1 silencing affects stress response-related symptoms in tomatoes [2]. In addition, SlGAME5 mediated shifts in steroidal alkaloid content during tomato evolution; it also represents the catalytic enzyme responsible for the last step in the transformation of tomatidine into non-toxic esculeoside A [14,16]. Potatoes (*Solanaceae* family) also contain an abundance of SAs [3]. Three GTs involved in the biosynthesis of a-solanine and a-chaconine (from the aglycone solanidine) have been identified: SGT1, SGT2, and SGT3 [33]. The primary glycosyl unit of SGT1 includes Gal and Glc, while for SGT2, it is Glc, and for SGT3, it is a UDP-Rha: b-solanine/b-chaconine rhamnosyltransferase [34,35]. 

GAME family genes belong to the GT family, which can affect the content of toxic substances and plant toxicity by catalyzing the enzymatic reaction of multiple substrates combined with a variety of donors. In this study, it was confirmed that SlGAME5-like can catalyze the glycosylation of lycopene, kaempferol and quercetin using UDP-glucose and UDP-galactose as substrates. Different from the known typical catalytic lycoposide glycosylation gene SlGAME1, SlGAME1 mainly targets lycoposylation in leaves, while SlGAME5 and SlGAME5-like mainly catalyze lycoposide and flavonol in fruits, thus improving tomato fruit flavor (Figure 3a,b; Figure S7). Thus, the GAME family gene has a powerful function. The transformation of toxic substances into non-bitter and non-toxic substances during tomato ripening is also a complex regulatory process that has not been clearly analyzed. In this study, the research on the function of the SlGAME5-like gene also provides new ideas for the future regulation of SA and SGAs in tomatoes and the cultivation of new tomato varieties with low SGA contents.

### 3.3. SlDOG1 Mediates GAMEs to Regulation of SA and SGA Biosynthesis 

Steroidal alkaloids are major (and secondary) metabolites in *Solanum* species, with more than 20,000 SAs reported across thousands of species to date [36]. Previous research on SGAs has focused on elucidating their structure and composition across species, as well as characterizing relevant biosynthetic pathways; however, the molecular mechanisms regulating steroidal alkaloid metabolism remain unknown. In tomatoes, SlGAME9 represents an AP2/ERF-type transcription factor that regulates the biosynthesis of SGAs [13]. Furthermore, SlGAME9 co-binds with the SlMYC2 transcription factor to also regulate SGA biosynthesis genes [13]. In addition, SlERF.D6 can not only promote fruit ripening as AP2/ERF family transcription factors but also regulate the expression of genes in several SGAs synthesis pathways [37]. In this study, we identified a SlDOG1-like protein using GWAS and mGWAS. Genetic and biochemical assays showed that SlDOG1 binds the promoters of *SlGAME5*, *SlGAME5-like*, and *SlGAME1*, activating *SlGAME5*, *SlGAME5-like*, and *SlGAME1* expression. SlDOG1-OE lines revealed that SlDOG1 promotes hydroxytomatine, lycoperside B, and (lycoperside H) FA accumulation in tomatoes (Figure 4g). The functions of the DOG1 gene have been reported in other species, such as seed dormancy and delayed flowering. At present, no scientists have investigated the function of DOG1 in regulating the SGA synthesis pathway. These findings provide insight into the transcriptional regulation of SGAs in *Solanaceae* plants, as well as a basis for engineering these antinutritional compounds in plants.

### 3.4. Favorable Natural Variation Provides the Basis for Tomato Breeding

The breeding history of tomatoes mainly experienced four stages: domestication, improvement, differentiation, and introgression. Domestication is the first stage of plant breeding. In the early stages of tomato domestication, preferences for fruit taste and weight drove the artificial selection of tomato crops [23,29,38]. However, in the process of selection for fruit weight, metabolism-related genes linked to those controlling fruit weight experienced hitchhiking effects and tended to be co-inherited [39]. As tomato fruits ripen, tomatidine is gradually converted to esculeoside A (which is less toxic), and changes in flavor characteristics also occur. Therefore, in the process of breeding, human beings selected for the size of the tomato fruit while other traits were selected, which gradually reduced the bitterness and toxic compounds in tomato fruit, thus leading to a change in tomato fruit flavor [16]. Here, we identified a DOG1-like family transcription factor; within this locus, highly significant polymorphisms were identified across three subsets of accessions (Figure 5). For example, a non-synonymous A-to-G substitution underlies variation in steroidal alkaloid content among tomato varieties (Figure 5d). Moreover, the frequency of the G base in the population gradually increased, indicating that in the breeding process, G base materials may be favored by consumers and selected by breeders because of the low content of bitter substances in them. In other words, *SlDOG1* encodes different protein structures in different varieties, in turn affecting the content of steroidal alkaloids in tomatoes. In the process of tomato domestication and improvement, the tomatidine content (as regulated by *SlDOG1*) gradually decreased as fruits became larger and their taste improved. As such, relevant natural variation could be used (e.g., via gene editing) to affect changes in crop SA contents, fruit quality, and flavor. However, more data are needed to explore this possibility, for example, via the testing of genetic materials with different mutations.

## 4. Materials and Methods

### 4.1. Plant Material and Growth Conditions

Tomato (Micro-Tom) plants and *Nicotiana benthamiana* were grown in a greenhouse at 24 °C under a 16/8 h light/dark at Hainan University, Haikou, China. Samples of tomato roots, stems, leaves, flowers, green fruits, and ripe (red) fruits were collected and quickly stored at −80 °C.

### 4.2. Sample Extraction and Analysis

Plant tissue samples of 0.1 g were extracted with 1.0 mL 70% methanol by sonication at room temperature for 10 min, followed by centrifugation at 5500× *g* for 10 min. The supernatants were filtered through a 0.22 μm membrane. SGAs and SAs were analyzed according to previously described methods [16].

### 4.3. Plant Tissue Total RNA Extraction and RT-qPCR

Total RNA was extracted from the ground samples using TRIzol (Vazyme; Transgen Biotechnology Co., Ltd., Beijing, China). Tomato cDNA was then obtained by reverse transcription of 2 μg of total RNA using a one-step reverse transcription kit (TransGen Biotech; Dalian, China). RT-qPCR was performed with SYBR Green PCR master mix (Takara; Dalian, China) on an Agilent AriaMx real-time fluorescent quantitative PCR system; the 2^−ΔΔCT^ calculation method was used for analysis, and all gene expression levels were normalized using an internal reference gene (Solyc11g005330). The primers are listed in Supplemental Data S1.

### 4.4. Phylogenetic Analysis

The amino acid sequence (and related sequences) of tomato UGT was obtained from the *Solanaceae* database (https://solgenomics.net/; accessed on 7 April 2022), and the amino acid sequence of rice UGT was obtained from the National Rice Database of China (https://www.ricedata.cn/; accessed on 7 April 2022); for related species, UGT sequences were obtained from the NCBI Database (https://www.ncbi.nlm.nih.gov/; accessed on 7 April 2022). Next, the amino acid sequences were aligned using MUSCLE (Multiple Protein Sequence Alignment) in MEGA-7.0, and a phylogenetic tree was constructed using the neighbor-joining method. All parameters were set to default values, and the phylogenetic analysis was reliable as evaluated using 1000 bootstrap replicates. Sequence information is available in Supplemental Data S2.

### 4.5. Vector Construction and Generation of Transgenic Lines

A transgenic vector was used for the Agrobacterium-mediated transformation of tomato plants. Golden gate cloning technology was used to obtain a PBI-121 vector. The plasmid with the correct insertion was introduced into Agrobacterium tumefaciens strain LBA4404, and tomato transformation was carried out as described previously (Ying et al., 2020) [40]. Totipotent plant cells developed into transgenic tomato plants in about 3–4 months.

### 4.6. Subcellular Localization

The specific location of a protein of interest was then observed in tobacco (*Nicotiana benthamiana*) leaf cells. Green fluorescent protein (GFP) was used to mark the target gene and acted as a reporter protein, while red fluorescent protein (RFP) was fused to the localization protein for use as a marker. In this study, all three genes were predicted to be located in the nucleus, so a gene with known nuclear localization, Ghd7, was selected as a red fluorescent protein marker (Xue et al., 2008) [41]. The gene sequence was amplified from Micro-Tom cDNA using gene-specific primers, and the GFP fusion protein vector was constructed using Gateway technology. The fusion protein vector was transformed into GV3101 Agrobacterium-competent cells according to the manufacturer’s instructions (Weidi Biotechnology). Positive colonies were then picked to co-transfect GFP and RFP to the tobacco leaf epidermis; these were cultured in the dark for 24 h and in the light for 12 h. Visualization was performed using a Zeiss utility LSM 800 laser confocal microscope.

### 4.7. Dual-Luciferase Reporter Gene

A high-fidelity amplification enzyme (TOYOBO) was used to amplify promoter and transcription factor sequences from the Micro-Tom genome via PCR. Using Gateway technology, the promoter sequence was inserted upstream of the luciferase gene to obtain a LUC reporter vector for the promoter, and the transcription factor sequence was inserted downstream of luciferase to obtain a LUC reporter vector for the transcription factor. For the dual-luciferase reporter experiments, the transcription factor expression plasmid and the reporter gene plasmid were used to co-transform tobacco leaf epidermal cells, which were cultured in the dark for 24 h and then in the light for 16 h. Luciferase activity assays were performed using the Promega GLOMAX reporter system. 

### 4.8. Yeast One-Hybrid System

The complete transcription factor sequence was amplified from tomato cDNA using gene-specific primers and inserted into a pGADT7 vector; similarly, the promoter of the structural gene was amplified from the tomato genome and inserted into a pHIS2 vector to form a recombinant vector. The recombinant vector was co-transformed into Y187 competent cells according to the manufacturer’s instructions (Shanghai Weidi Biotechnology), while the pGADT7 empty vector was transformed as a negative control. Positive strains were screened using auxotrophic yeast medium SD/-Leu/-Trp before culturing in auxotrophic yeast medium SD/-Leu/-Trp/-His with different concentrations of 3-AT (3-amino-1,2,4-triazole). Interactions were assessed according to colony morphology and status.

### 4.9. Enzyme Kinetics

The kinetic constants of glycosyltransferase were determined for flavonoid and tomatidine compounds. The activity was determined with 0–200 μM quercetin and tomatine, and a fixed concentration of 15 mM UDP-galactose was used as the glycosyl donor [42]. 

### 4.10. Genome-Wide Association Analysis 

Whole-genome sequencing (WGS) sequence data were obtained from previous studies [16,29]. SNP detection and filtering have been described previously [43,44,45,46]. A total of 11606972 SNPs were used in GWAS for different subgroups of tomato, *S. pimpinellifolium* (PIM), *S. lycopersicum* var. *cerasiformeand* (CER) and *S. lycopersicum* var. *Lycopersicum* (BIG) [29]. The GWAS was carried out using EMMAX [47]. Because of the non-independence of SNPs (due to strong LD), thresholds for significant association detection are usually too strict when derived from the total number of markers. The number of independent markers (3,846,762) was therefore determined using GEC [48] and a suggestive threshold was set for the association mapping panel (*p* < 2.60 × 10^−7^, or 1/3,846,762) to control the type I error rate. Genomic regions associated with metabolite traits were identified as previously described [49]. The linkage disequilibrium method has been described previously [50].

### 4.11. Identification of Domestication and Improvement Sweeps 

Vcftools (version 0.1.16) [51] was used to measure the diversity of genetic information (π) in 50-kb windows with a step size of 5 kb for wild accessions of S. *pimpinellifolium* (PIM), domesticated accessions of *S. lycopersicum* var. cerasiforme (CER), and improved accessions of *S. lycopersicum* (BIG). There were significant differences in nucleotide polymorphisms in genomic regions affected in domesticated accessions (πBIG) and improved accessions (πCER) versus wild accessions (πPIM). Windows with the top 5% highest ratios of πCER/πBIG (≥7.75) or πPIM/πCER (≥2.98) were selected as candidate domestication sweeps.

## 5. Conclusions

This manuscript attempts to understand the molecular mechanisms regulating SA and SGA formation. Here, a SlGAME5-like glycosyltransferase and the transcription factor SlDOG1 were identified. SlGAME5-like performed glycosidation in SAs and flavonol pathways in vitro. Furthermore, assessments of natural variation combined with functional analyses identified SlDOG1 as a major determinant of tomato SGA content, which also promoted SA and SGA accumulation via the regulation of the expression of GAME genes.

## Data Availability

The datasets presented in this study can be found in online repositories. The names of the repository/repositories and accession number(s) can be found below: National Center for Biotechnology Information (NCBI) BioProject database under accession number PRJNA783378.

## Supplementary Materials

The following supporting information can be downloaded at: https://www.mdpi.com/article/10.3390/ijms24043360/s1.
