# Correction: Zhao et al. The *SlDOG1* Affect Biosynthesis of Steroidal Glycoalkaloids by Regulating GAME Expression in Tomato. *Int. J. Mol. Sci.* 2023, *24*, 3360

**DOI:** 10.3390/ijms26030880

**Published:** 2025-01-21

**Authors:** Xuecheng Zhao, Yueran Zhang, Jun Lai, Yuan Deng, Yingchen Hao, Shouchuang Wang, Jun Yang

**Affiliations:** 1 Hainan Yazhou Bay Seed Laboratory, Sanya Nanfan Research Institute of Hainan University, Sanya 572025, China; 2 College of Tropical Crops, Hainan University, Haikou 570228, China

In the original publication [1], there was a mistake in Figure 4c as published. Upon carefully examining our original experimental results, we realized that due to our oversight, the image for the -Leu/-Trp result of *Pro SlGAME5*+SlDOG1 combination was duplicated twice, resulting in the image for the *Pro SlGAME5-like*+EV combination being identical to that of the *Pro SlGAME5*+SlDOG1 result. In the original publication, the image for *Pro SlGAME5-like*+SlDOG1 was actually for *Pro SlGAME5-like*+EV, while the actual image for the *Pro SlGAME5-like*+SlDOG1 combination was not included in the published paper.

We have now rectified this mistake by replacing the incorrect image for the actual *Pro SlGAME5-like*+EV result, and we have included the original experimental result for *Pro SlGAME5-like*+SlDOG1. It is important to emphasize that this correction does not affect the conclusion of our experiment. All vector combinations were successfully cotransformed into the yeast strain and grew normally on the -Leu/-Trp media, indicating a successful transformation.

The corrected Figure 4 appears below. The authors state that the scientific conclusions are unaffected. The authors apologize for any inconvenience caused and state that the scientific conclusions are unaffected. This correction was approved by the Academic Editor. The original publication has also been updated.

## Figures and Tables

**Figure 4:**
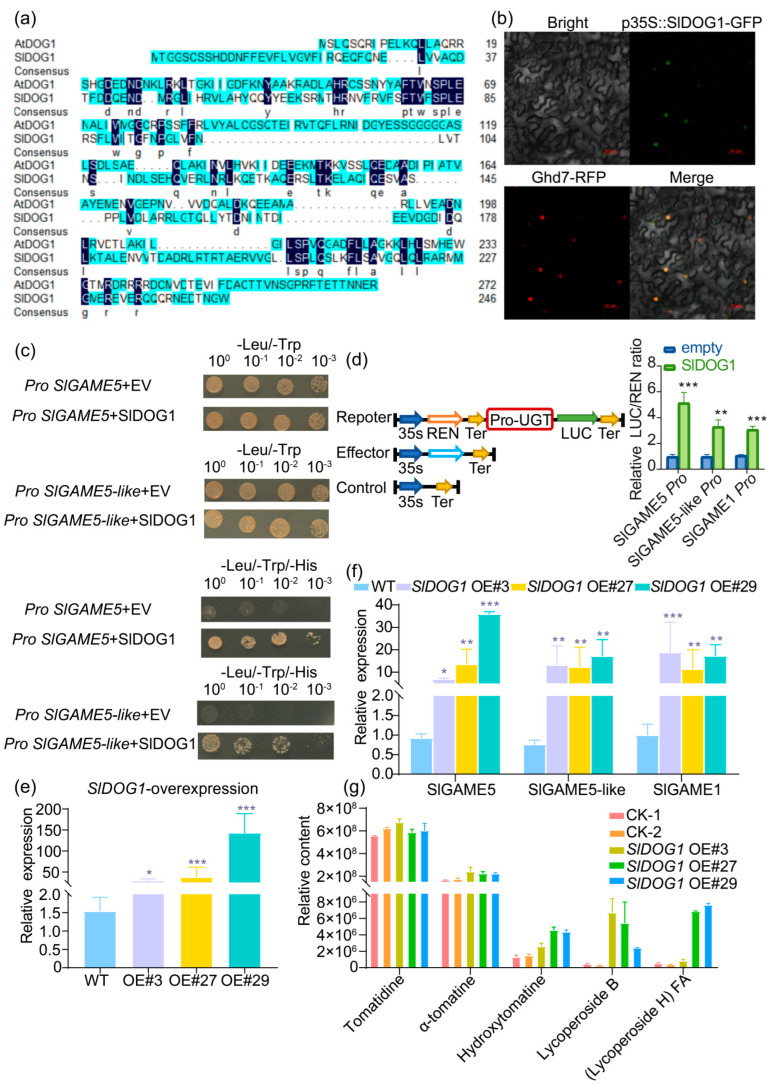
SlDOG1 interacts with SlGAME in vivo and in vitro. (**a**) Multiple sequence alignment of SlDOG1 and its homologs. (**b**) Nuclear localization of GFP-SlDOG1 fusion proteins in the leaf epidermal cells of *Nicotiana benthamiana*. Bar = 50 µm. (**c**) A yeast one-hybrid assay showing interactions between SlDOG1 and *SlGAME5*, *SlGAME5-like*. (**d**) Dual luciferase reporter assay showing how SlDOG1 can induce the activity of a common promoter of *SlGAME1-like* genes. (**e**) *SlDOG1* expression levels in three independent transgenic lines as determined by real-time PCR analysis. (**f**) The expression levels of *SlGAME5*, *SlGAME5-like*, and *SlGAME1* in SlDOG1-overexpressing plants as determined via real-time PCR. (**g**) SlDOG1-OE plants showed heightened accumulation of lycopene and its precursor. The absolute concentrations of both were determined in fully expanded leaves from four-week-old seedlings using UPLC-qTOF-MS and compared with those of wild-type plants of the same age. CK-1, CK-2: wild-type plants of the same age. Data were from three independent experiments and expressed as means ± SD. (n = 3). The differences were analyzed in two tailed comparisons with the control, and * *p* < 0.05; ** *p* < 0.01; *** *p* < 0.001 in the student’s *t*-test.
