# Supplementary material for: The SlDOG1 Affect Biosynthesis of Steroidal Glycoalkaloids by Regulating GAME Expression in Tomato
Source: Int J Mol Sci. 2023 Feb 8;24(4):3360. doi: 10.3390/ijms24043360 (PMC9960814; doi:10.3390/ijms24043360)
Supplement: Supplementary file 1 [file ijms-24-03360-s001.zip › supporting information.pdf]

Supporting information

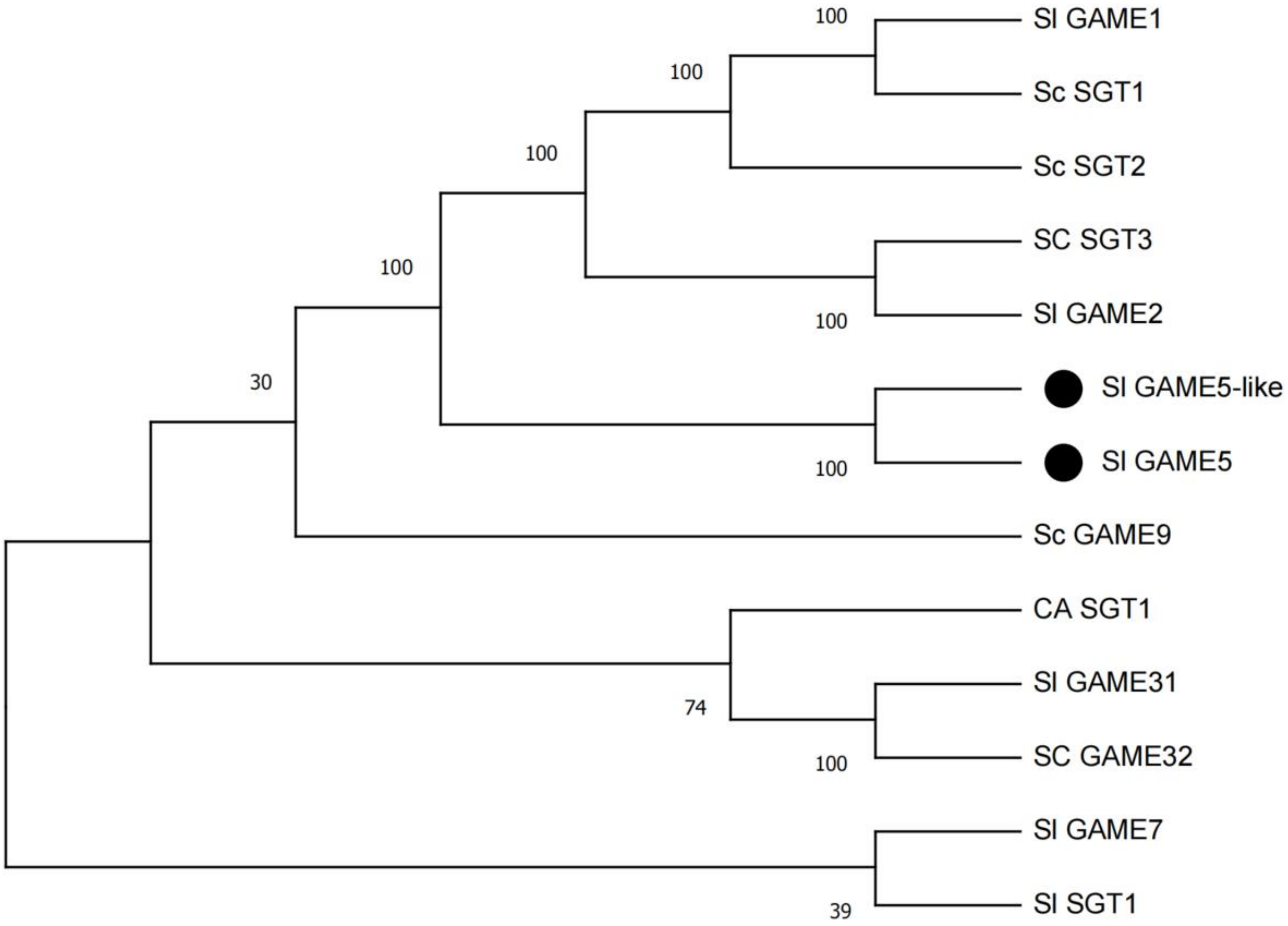

Fig S1. A phylogenetic tree resulting from MEGA analysis using amino acid sequences of GAME5 gene and other GAME members

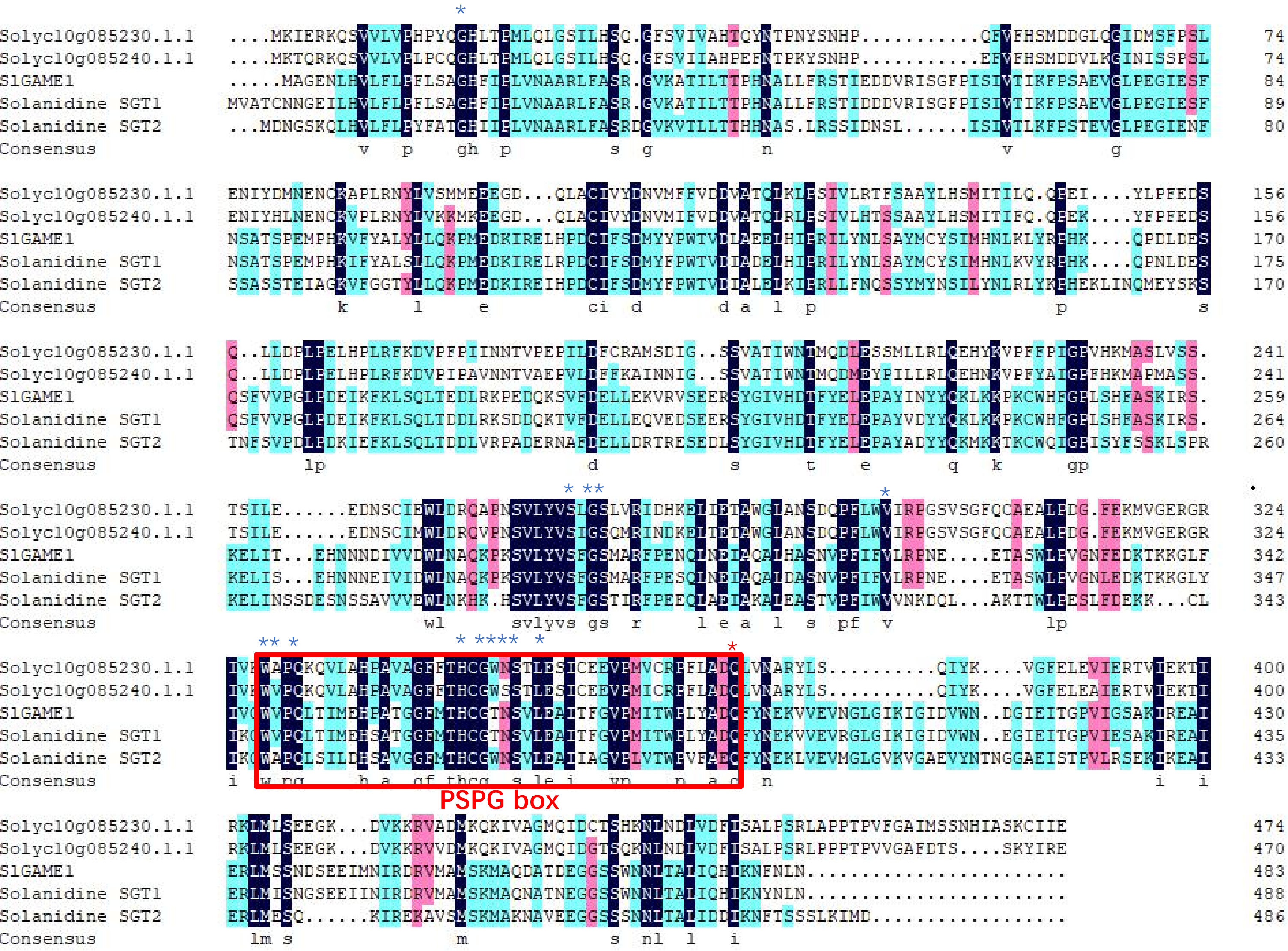

Fig S2. Amino acid sequence alignment of SlGAME5 and SlGAME5-like and three homologies.

The identified SlGAME5 and SlGAME5-like in multiple alignment with SlGAME1, SGT1 and SGT2.

The multiple alignment was performed using DNAMAN software. Red dots indicate amino acid residues that are involved in ligand binding.

Blue asterisks indicate UDP-sugar donor active sites Red rectangles indicate PSPG box

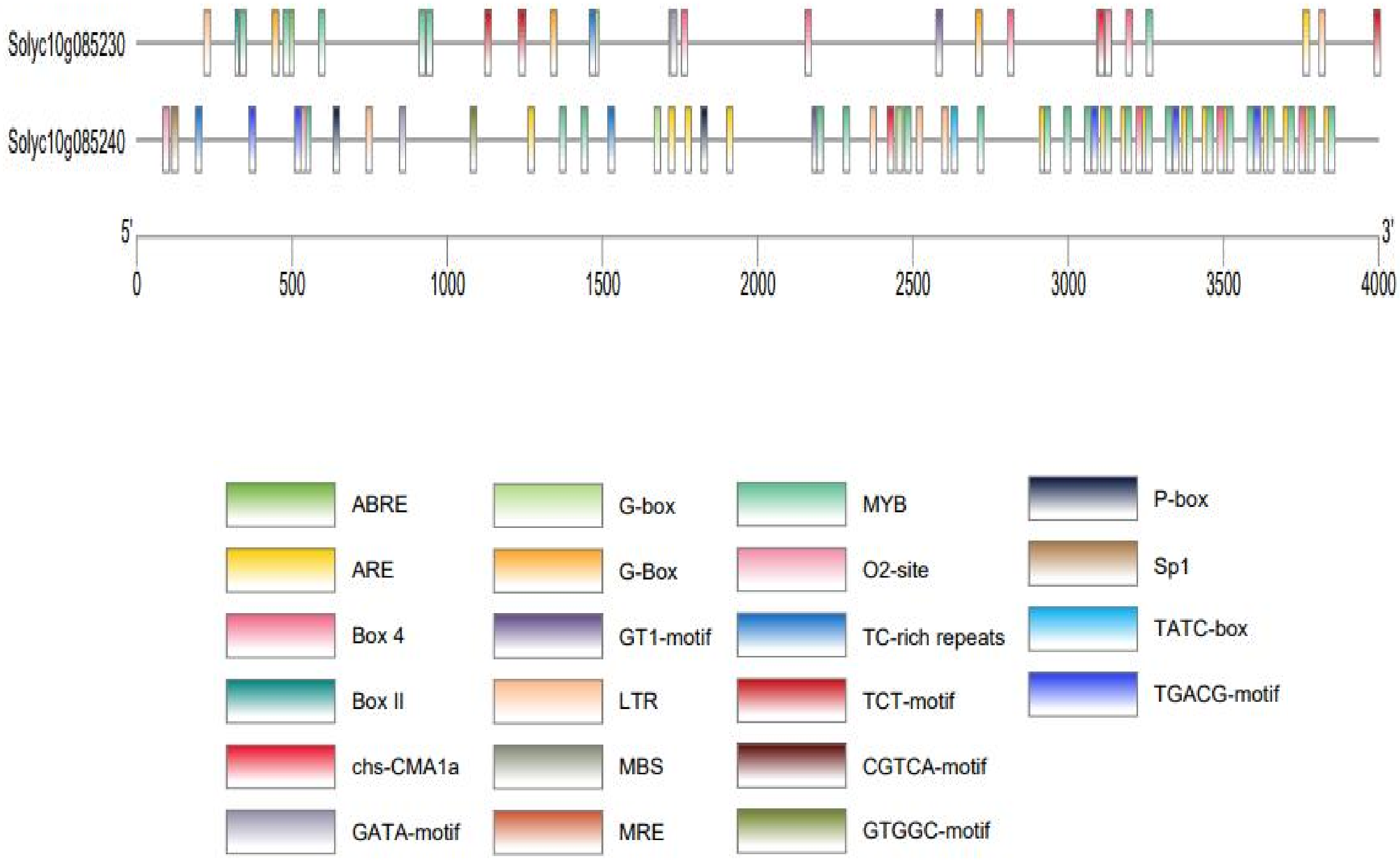

Fig S3. Gene structure and promoter cis-regulatory element analyses of tomato rSlGAME5 and rSlGAME5-like . Putative cis-elements in the promoter region of rSlGAME5 and rSlGAME5-like were predicted by PlantCARE.

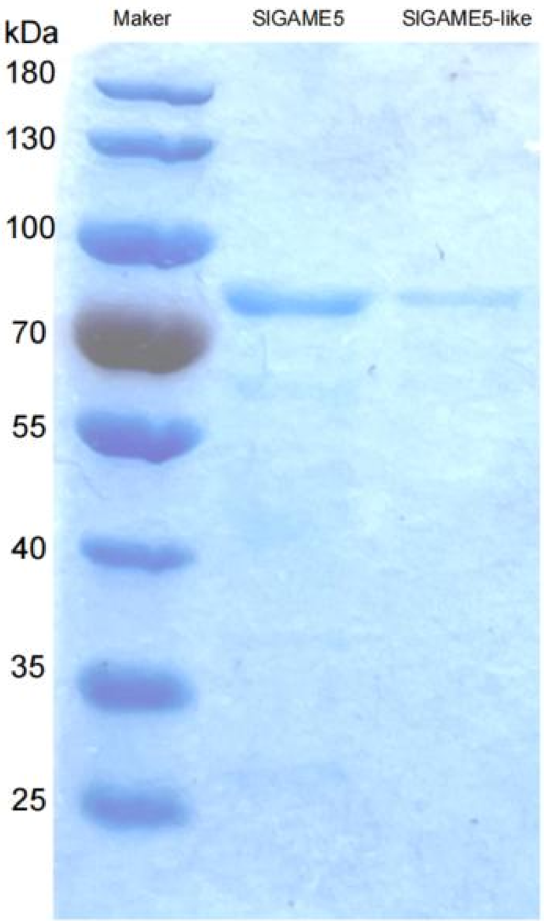

Fig S4. SDS-PAGE of rSlGAME5 and rSlGAME5-like protein fractions

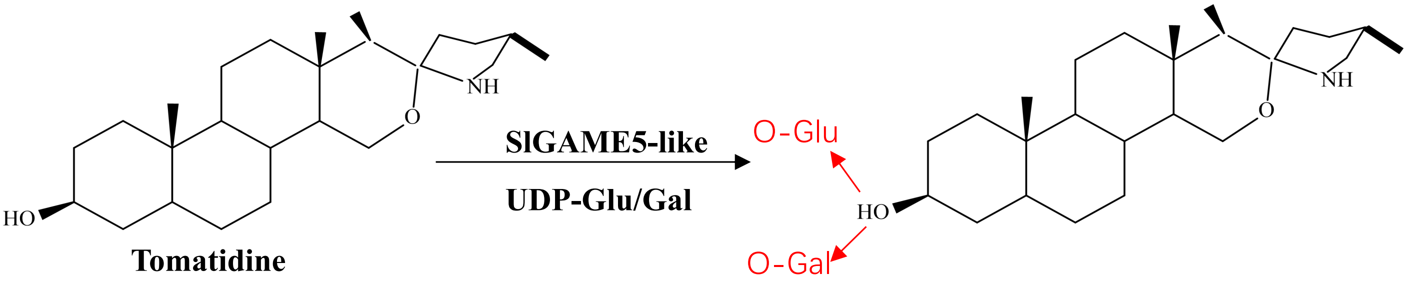

Fig S5. Catalytic reaction of rSlGAME5 and rSlGAME5-like

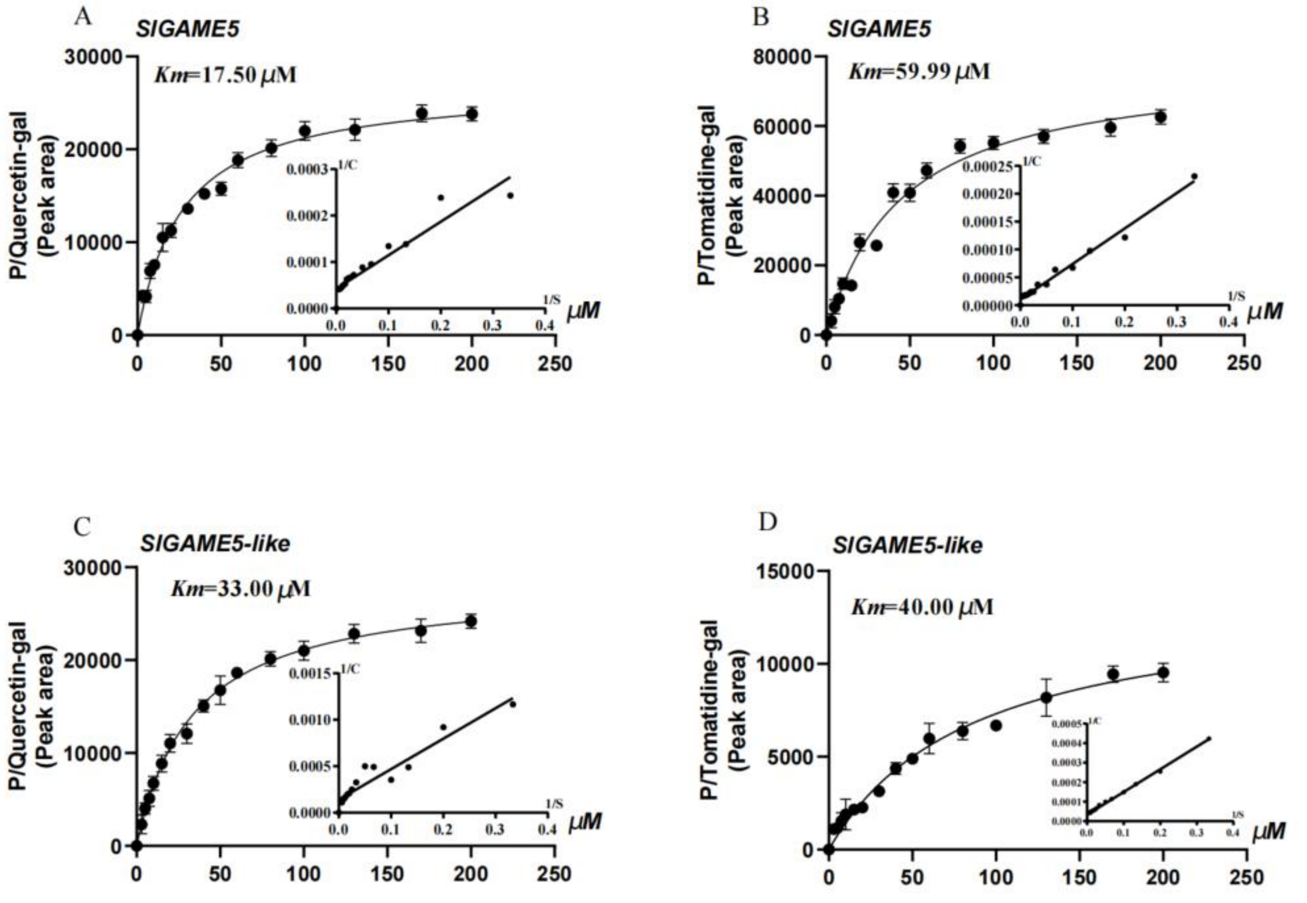

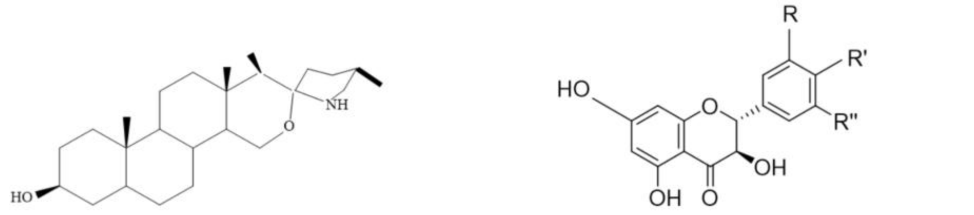

Tomatidine

Kaempferol: R=OH, R'=H, R''=H
Quercetin: R=OH, R'=OH, R''=H
Myricetin: R=OH, R'=OH, R''=OH

Name	Substrate	Km (μM)	Vmax (PKat·mg^{-1})	Kcat (s^{-1})	Kcat/Km (s^{-1}·M^{-1})
SlGAME5	Quercetin	17.50	25000	11150	637.14
	Tomatidine	59.99	100000	44600	743.46
SlGAME5-like	Quercetin	33.00	10000	4430	134.24
	Tomatidine	40.00	33333	14767	369.175

Fig S6. (A−D) Kinetics of SlGAME5 and SlGAME5-like to substrates, including quercetin and tomatidine. Chemical structures of substrates. Kinetic parameters of the recombinant SlGAME5 and SlGAME5-like proteins with quercetin and tomatidine aglycones as acceptor substrates and UDP-glucose as the donor substrate. Values represent the means ± SD from triplicate enzymatic assays.

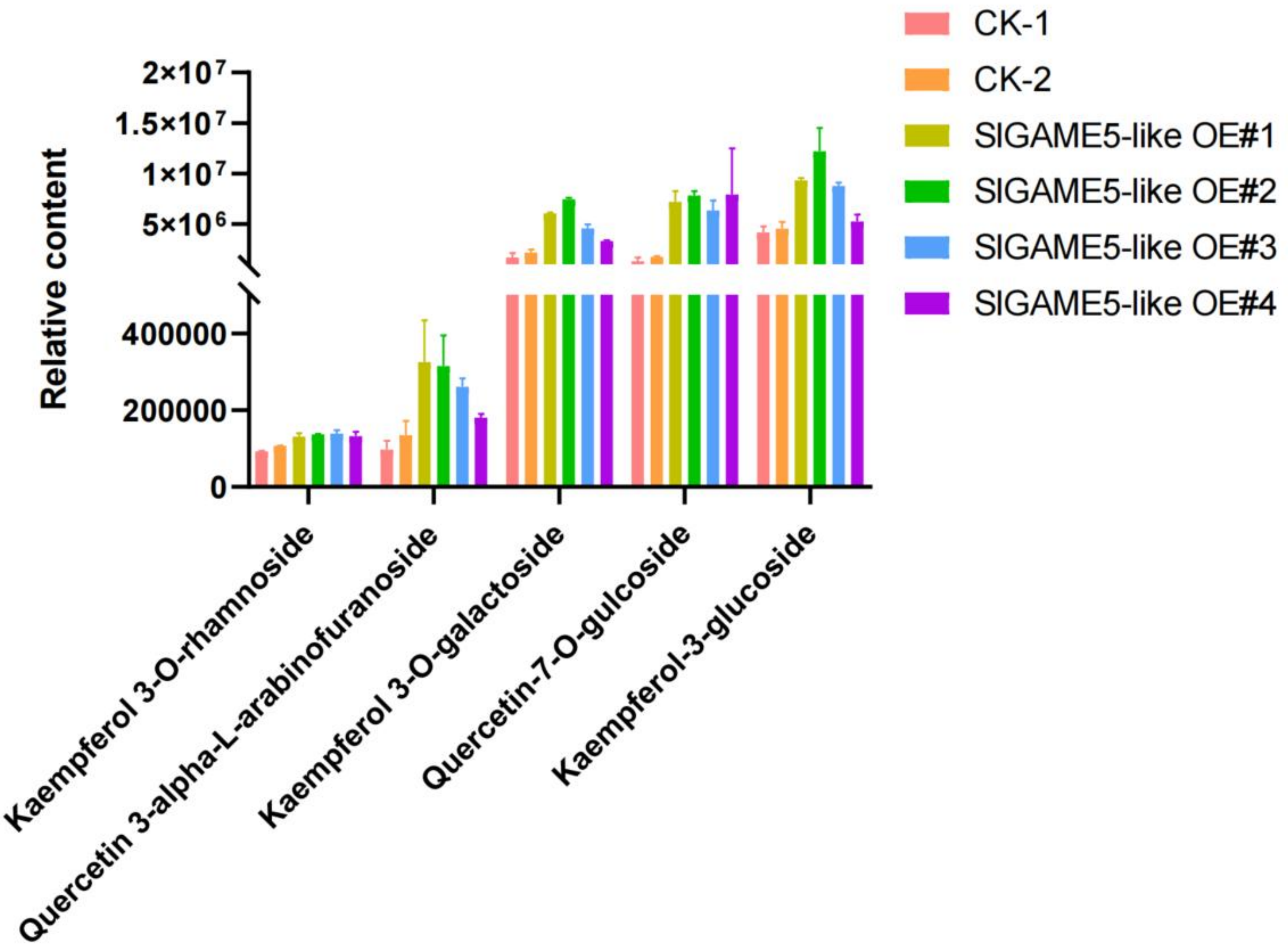

Fig S7. The content of flavonoids metabolites in the leaves of Sl-GAME5-like overexpressing plants was detected by LC-MS.

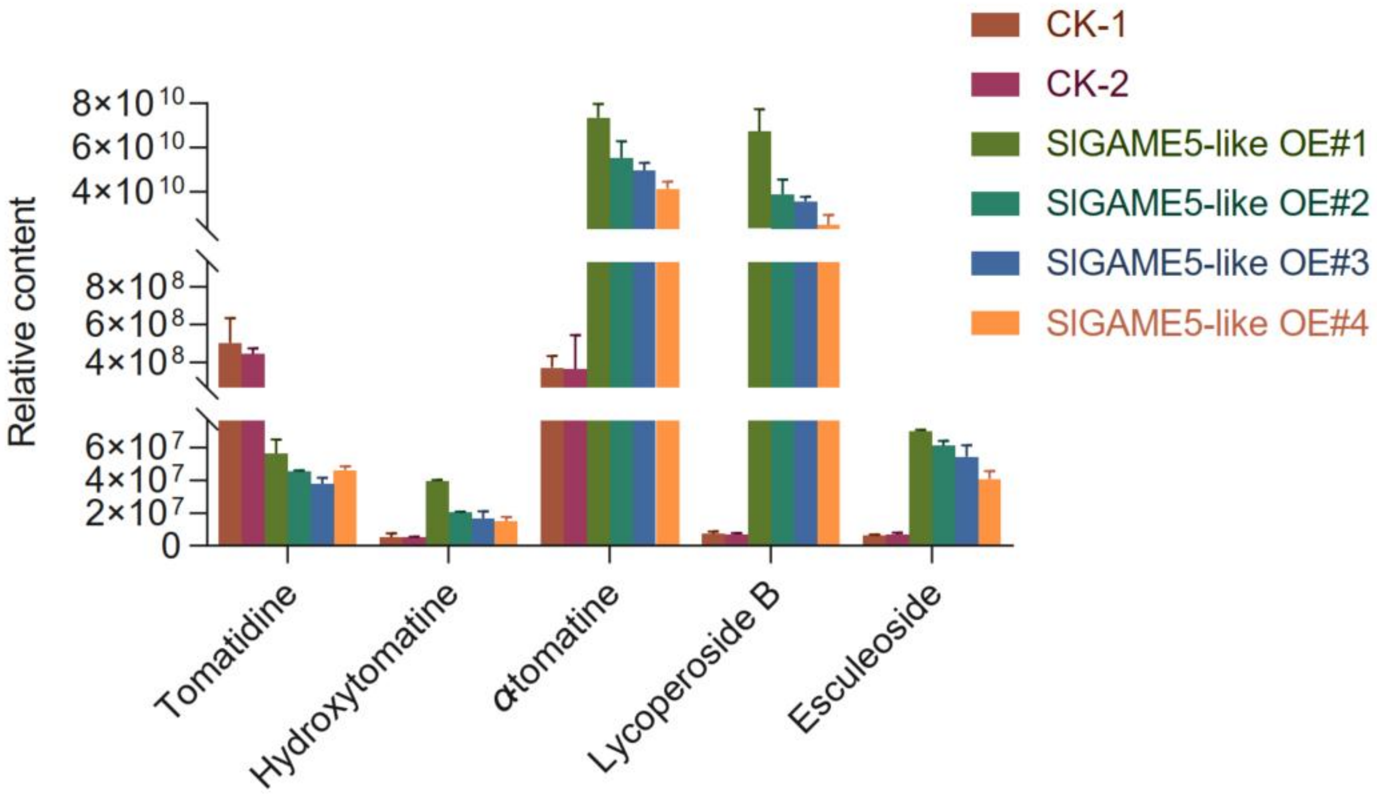

Fig S8. SlGAME5-like plants showed increased accumulation of lycopene and its precursor. The absolute concentrations of lycopene and its precursor were determined in breaker stage fruits using UPLC-qTOF-MS and compared with those of wild-type plants of the same age.

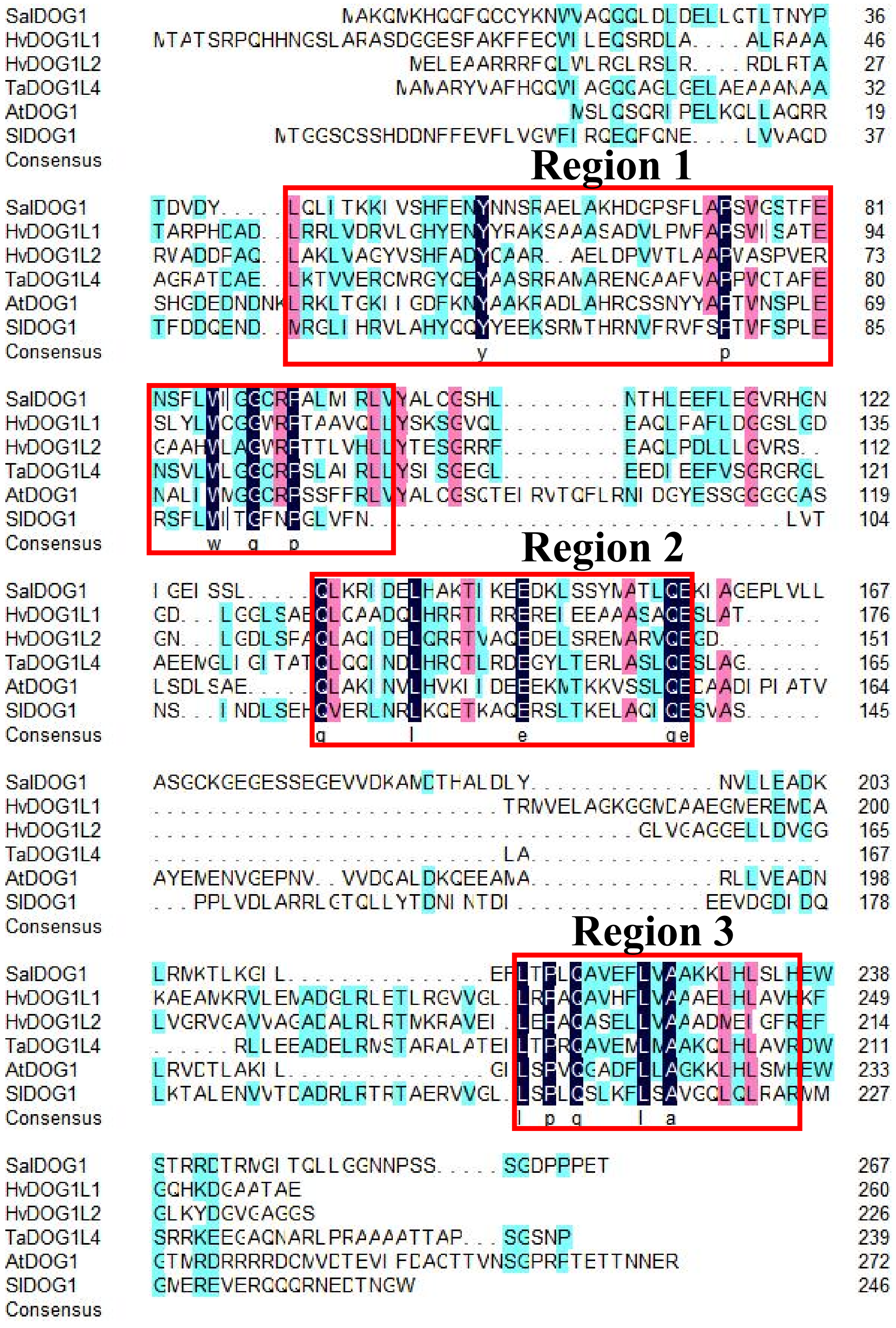

Fig S9. Multiple sequence alignment of SlDOG1 and its homologs.Different colors represent different degrees of similarity.

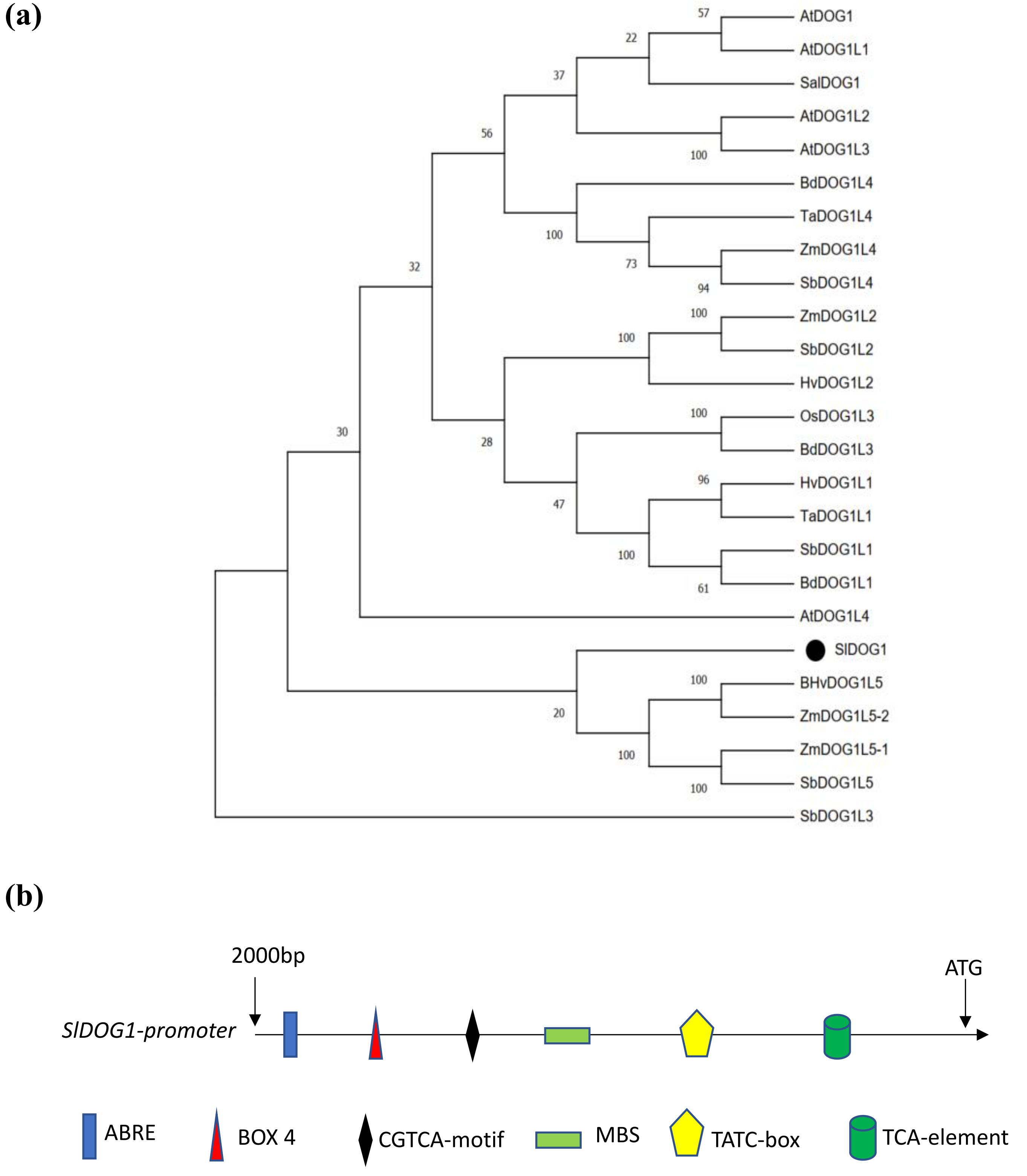

Fig S10. (a): Phylogenetic tree analyses of tomato SlDOG1. (b): Putative cis-elements in the promoter region of SlDOG1 was predicted by PlantCARE.

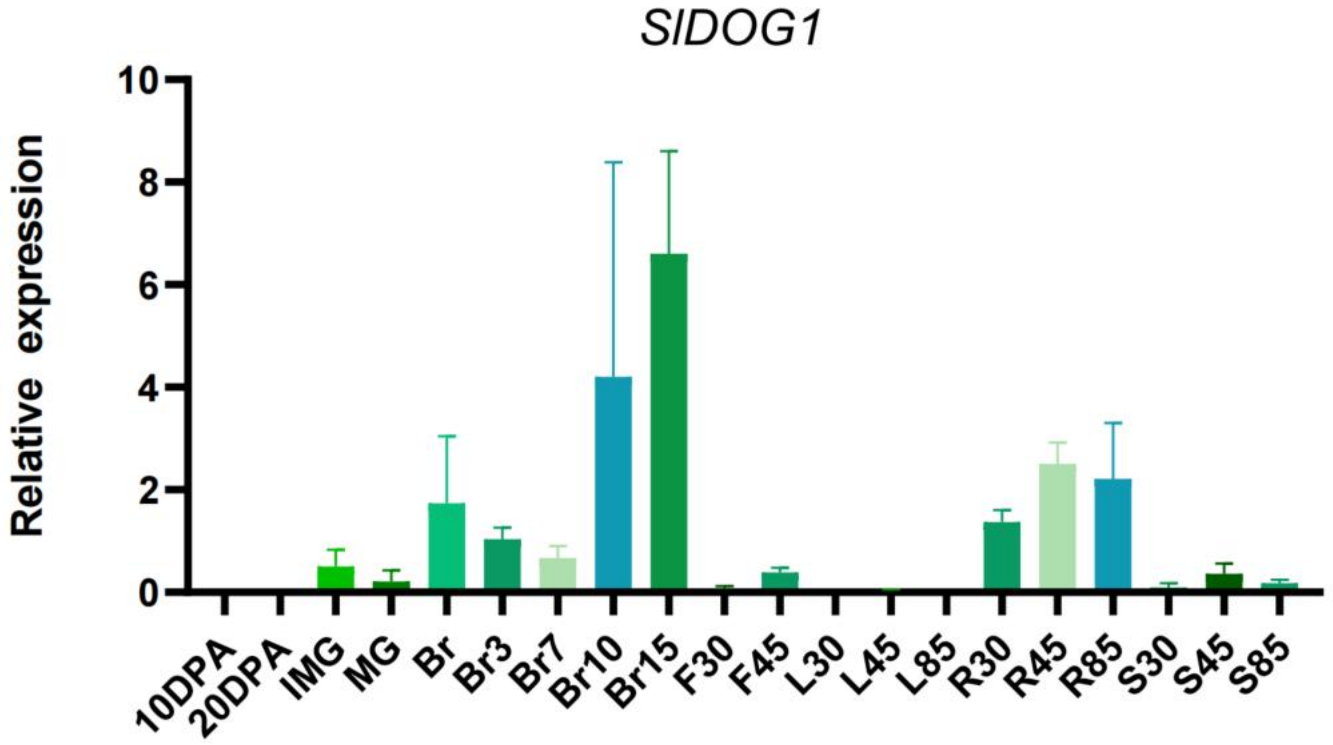

Fig S11. Expression of SlDOG1 in 20 different tissues and developmental stages of tomato.

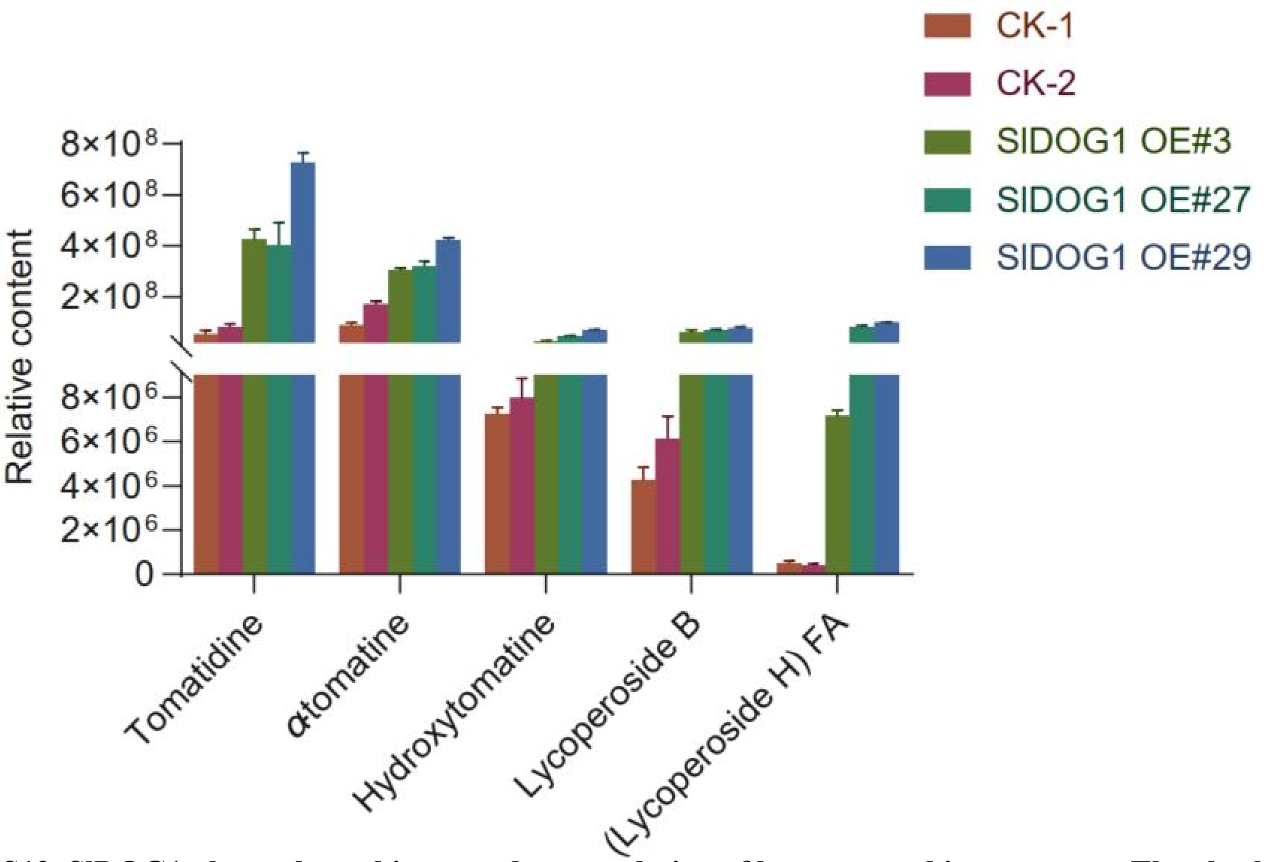

Fig S12. SlDOG1 plants showed increased accumulation of lycopene and its precursor. The absolute concentrations of lycopene and its precursor were determined in breaker stage fruits using UPLC-qTOF-MS and compared with those of wild-type plants of the same age.

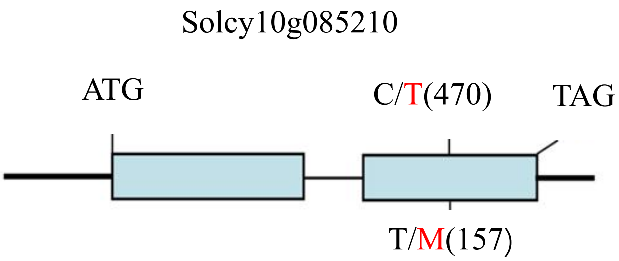

Fig S13. Point mutation diagram of SlDOG1.
